# Supplementary material for: Upstream open reading frames buffer translational variability during Drosophila evolution and development
Source: eLife. 2025 Jun 6;14:RP104074. doi: 10.7554/eLife.104074 (PMC12143884; doi:10.7554/eLife.104074)
Supplement: Supplementary file 1. [file elife-104074-supp1.docx]

**Supplementary File 1. Parameters used in our simulation**

| **Symbol** | **Description** | **Value** |
| --- | --- | --- |
| *Len_a* | Length of 5’-leader before uORF | 50 triplets |
| *Len_u* | Length of uORF | Default: 30 triplets  Variable: 2, 10, 20, 30, 40, 50, 100 triplets |
| *Len_b* | Distance between uORF and CDS | 50 triplets |
| *Len_m* | Length of CDS | 500 triplets |
| *Len_c* | Length of 3’UTR | 50 triplets |
| $\nu_{s}$ | Probability of movement of a 40S ribosome to the next position in a single action | 0.3* |
| $\nu_{Eu}$ | Probability of movement of an 80S ribosome to the next position in uORF in a single action | 0.3** |
| $\nu_{EC}$ | Probability of movement of an 80S ribosome to the next position in CDS in a single action | 0.5*** |
| *R_in_* | Probability of loading a new 40S ribosome at the 5’-terminus of the mRNA in a single action | 1000 values generated from uniform distribution or exponential distribution |
| $K_{up}$ | Dissociation probability of upstream 40S ribosome colliding with downstream 80S ribosome | 0 for downstream dissociation;  1 for upstream or double dissociation |
| $K_{d\mathrm{own}}$ | Dissociation probability of downstream 40S ribosome colliding with upstream 80S ribosome | 0 for upstream dissociation;  1 for downstream or double dissociation |
| $I_{uORF}$ | Probability of translation initiation at the uORF start codon in a single action | Single-uORF model:  0, 0.1, 0.2, 0.3, 0.4, 0.5  Double-uORF model:  0, 0.1, 0.2, 0.3, 0.4 |
| $I_{CDS}$ | Probability of translation initiation at the CDS start codon in a single action | Single-uORF model:  0.1, 0.2, 0.3, 0.4, 0.5, 0.6, 0.7, 0.8, 0.9, 1.0  Double-uORF model:  0.9 |

*Adopted from Andreev *et al.*’s original ICIER model (Andreev et al., 2018), corresponding to a movement rate of 5 triplets/s.

**Adopted from Andreev *et al.*’s original ICIER model (Andreev et al., 2018), corresponding to a movement rate of 5 codons/s.

*** Considering that uORFs usually encode blocking peptides (Ivanov et al., 2018; Lovett and Rogers, 1996; Luo and Sachs, 1996; Raney et al., 2002; Vilela and McCarthy, 2003) or contain stalling codons (Bottorff et al., 2022; Lin et al., 2019; Meijer and Thomas, 2003), we set $\nu_{Eu}$ slightly lower than $\nu_{EC}$.
