# Supplementary material for: Upstream open reading frames buffer translational variability during Drosophila evolution and development
Source: eLife. 2025 Jun 6;14:RP104074. doi: 10.7554/eLife.104074 (PMC12143884; doi:10.7554/eLife.104074)
Supplement: Supplementary file 2. [file elife-104074-supp2.docx]

**Supplementary File 2. Mapping statistics of Ribo-Seq and matched mRNA-Seq libraries**

| **Sample** | **Species*** | **Library type** | | **Total reads (M)** | **After quality control (M) **** | | **Unique mapping rate (%)** | **Multiple mapping rate (%)** |
| --- | --- | --- | --- | --- | --- | --- | --- | --- |
| **0-2 h embryos** | *D. melanogaster* | mRNA-Seq | | 61.01 | | 52.74 | 93.14 | 2.84 |
| **2-6 h embryos** | *D. melanogaster* | mRNA-Seq | | 59.44 | | 52.07 | 93.26 | 3.24 |
| **6-12 h embryos** | *D. melanogaster* | mRNA-Seq | | 53.56 | | 45.11 | 92.33 | 3.09 |
| **12-24 h embryos** | *D. melanogaster* | mRNA-Seq | | 61.18 | | 50.64 | 91.89 | 4.31 |
| **Third-instar larvae** | *D. melanogaster* | mRNA-Seq | | 58.28 | | 45.46 | 88.53 | 6.09 |
| **P7-8 pupae** | *D. melanogaster* | mRNA-Seq | | 52.99 | | 41.16 | 92.65 | 4.46 |
| **Female adult heads** | *D. melanogaster* | mRNA-Seq | | 21.48 | | 16.87 | 93.00 | 3.27 |
| **Male adult heads** | *D. melanogaster* | mRNA-Seq | | 16.32 | | 14.18 | 91.17 | 4.24 |
| **Female adult bodies rep1** | *D. melanogaster* | mRNA-Seq | | 16.84 | | 11.20 | 88.83 | 5.76 |
| **Female adult bodies rep2** | *D. melanogaster* | mRNA-Seq | | 24.94 | | 19.37 | 88.73 | 6.00 |
| **Male adult bodies rep1** | *D. melanogaster* | mRNA-Seq | | 19.79 | | 16.01 | 91.62 | 6.39 |
| **Male adult bodies rep2** | *D. melanogaster* | mRNA-Seq | | 11.24 | | 7.64 | 89.86 | 6.69 |
| **0-2 h embryos** | *D. melanogaster* | Ribo-Seq | | 64.81 | | 51.96 | 90.36 | 6.31 |
| **2-6 h embryos** | *D. melanogaster* | Ribo-Seq | | 53.14 | | 41.19 | 69.47 | 16.92 |
| **6-12 h embryos** | *D. melanogaster* | Ribo-Seq | | 66.24 | | 46.76 | 86.02 | 8.94 |
| **12-24 h embryos** | *D. melanogaster* | Ribo-Seq | | 63.7 | | 47.45 | 88.96 | 6.30 |
| **Third-instar larvae** | *D. melanogaster* | Ribo-Seq | | 39.53 | | 22.13 | 58.83 | 6.82 |
| **P7-8 pupae** | *D. melanogaster* | Ribo-Seq | | 41.53 | | 20.33 | 80.92 | 5.51 |
| **Female adult heads** | *D. melanogaster* | Ribo-Seq | | 63.94 | | 39.90 | 94.39 | 2.92 |
| **Male adult heads** | *D. melanogaster* | Ribo-Seq | | 58.22 | | 23.87 | 93.36 | 3.24 |
| **Female adult bodies rep1** | *D. melanogaster* | Ribo-Seq | | 44.11 | | 19.85 | 84.20 | 6.36 |
| **Female adult bodies rep2** | *D. melanogaster* | Ribo-Seq | | 41.95 | | 18.73 | 70.74 | 11.33 |
| **Male adult bodies rep1** | *D. melanogaster* | Ribo-Seq | | 42.56 | | 18.98 | 79.39 | 8.65 |
| **Male adult bodies rep2** | *D. melanogaster* | Ribo-Seq | | 39.52 | | 28.66 | 81.19 | 6.93 |
| **0-2 h embryos** | *D. simulans* | mRNA-Seq | | 19.92 | | 18.33 | 90.85 | 5.82 |
| **2-6 h embryos** | *D. simulans* | mRNA-Seq | | 13.88 | | 7.47 | 88.32 | 7.46 |
| **6-12 h embryos** | *D. simulans* | mRNA-Seq | | 21.13 | | 18.64 | 89.26 | 7.06 |
| **12-24 h embryos** | *D. simulans* | mRNA-Seq | | 16.17 | | 13.96 | 87.77 | 8.47 |
| **Third-instar larvae** | *D. simulans* | mRNA-Seq | | 17.56 | | 12.69 | 89.88 | 6.57 |
| **P7-8 pupae** | *D. simulans* | mRNA-Seq | | 23.87 | | 19.04 | 89.92 | 5.62 |
| **Female adult heads** | *D. simulans* | mRNA-Seq | | 17.13 | | 15.11 | 89.70 | 6.39 |
| **Male adult heads** | *D. simulans* | mRNA-Seq | | 17.72 | | 15.96 | 90.73 | 6.15 |
| **Female adult bodies** | *D. simulans* | mRNA-Seq | | 21.64 | | 15.37 | 85.24 | 9.20 |
| **Male adult bodies** | *D. simulans* | mRNA-Seq | | 21.54 | | 17.34 | 87.45 | 8.84 |
| **0-2 h embryos** | *D. simulans* | Ribo-Seq | | 63.38 | | 49.48 | 80.09 | 10.81 |
| **2-6 h embryos** | *D. simulans* | Ribo-Seq | | 53.46 | | 38.09 | 77.27 | 13.72 |
| **6-12 h embryos** | *D. simulans* | Ribo-Seq | | 57.76 | | 43.84 | 81.58 | 10.81 |
| **12-24 h embryos** | *D. simulans* | Ribo-Seq | | 50.55 | | 35.05 | 83.55 | 8.21 |
| **Third-instar larvae** | *D. simulans* | Ribo-Seq | | 67.69 | | 35.40 | 86.44 | 7.10 |
| **P7-8 pupae** | *D. simulans* | Ribo-Seq | | 61.74 | | 40.45 | 87.45 | 4.76 |
| **Female adult heads** | *D. simulans* | Ribo-Seq | | 62.52 | | 26.18 | 86.51 | 4.58 |
| **Male adult heads** | *D. simulans* | Ribo-Seq | | 65.07 | | 28.99 | 89.62 | 3.96 |
| **Female adult bodies** | *D. simulans* | Ribo-Seq | | 59.47 | | 12.91 | 83.01 | 5.72 |
| **Male adult bodies** | *D. simulans* | Ribo-Seq | | 53.55 | | 18.35 | 87.44 | 5.61 |
|  |  |  |  | | |  |  |  |

* The mRNA-seq and Ribo-Seq datasets for *D. melanogaster* were previously generated from Zhang *et al.* (Hong Zhang et al., 2018). The datasets for *D. simulans* were generated in this study.

** After removing the reads mapped to rRNA, miscRNA (snoRNA, snRNA, rRNA, tRNA), the yeast genome, and the Wolbachia genome.
