# Supplementary material for: Upstream open reading frames buffer translational variability during Drosophila evolution and development
Source: eLife. 2025 Jun 6;14:RP104074. doi: 10.7554/eLife.104074 (PMC12143884; doi:10.7554/eLife.104074)
Supplement: Supplementary file 4. [file elife-104074-supp4.docx]

**Supplementary File 4.**

**Mapping statistics of mRNA-Seq libraries for *bcd* uKO2/uKO2 mutant and WT flies**

| **Sample** | **Condition** | **Stain** | **Stage** | **Total read-pairs (M)** | **Unique mapping reads (M)** | **Unique mapping rate (%)** | **Multiple mapping rate (%)** |
| --- | --- | --- | --- | --- | --- | --- | --- |
| **uKO2-0-2 h-embryo-29-rep1** | 29$℃$ | Mutant | 0-2 h | 19.39 | 16.73 | 86.31 | 9.13 |
| **uKO2-0-2 h-embryo-29-rep2** | 29℃ | Mutant | 0-2 h | 18.72 | 12.72 | 67.94 | 26.64 |
| **uKO2-2-6 h-embryo-29-rep1** | 29℃ | Mutant | 2-6 h | 19.60 | 16.76 | 85.51 | 11.60 |
| **uKO2-2-6 h-embryo-29-rep2** | 29℃ | Mutant | 2-6 h | 18.01 | 15.40 | 85.52 | 11.53 |
| **uKO2-6-12 h-embryo-29-rep1** | 29℃ | Mutant | 6-12 h | 18.55 | 16.26 | 87.64 | 7.52 |
| **uKO2-6-12 h-embryo-29-rep2** | 29℃ | Mutant | 6-12 h | 19.76 | 17.31 | 87.58 | 7.79 |
| **uKO2-12-24 h-embryo-29-rep1** | 29℃ | Mutant | 12-24 h | 17.48 | 15.23 | 87.14 | 8.36 |
| **uKO2-12-24 h-embryo-29-rep2** | 29℃ | Mutant | 12-24 h | 18.78 | 15.98 | 85.09 | 10.05 |
| **w1118-0-2 h-embryo-29-rep1** | 29℃ | WT | 0-2 h | 19.10 | 17.24 | 90.25 | 6.87 |
| **w1118-0-2 h-embryo-29-rep2** | 29℃ | WT | 0-2 h | 19.02 | 17.12 | 90.01 | 7.38 |
| **w1118-2-6 h-embryo-29-rep1** | 29℃ | WT | 2-6 h | 18.54 | 15.60 | 84.11 | 12.40 |
| **w1118-2-6 h-embryo-29-rep2** | 29℃ | WT | 2-6 h | 19.24 | 16.34 | 84.93 | 11.84 |
| **w1118-6-12 h-embryo-29-rep1** | 29℃ | WT | 6-12 h | 18.61 | 15.87 | 85.28 | 10.50 |
| **w1118-6-12 h-embryo-29-rep2** | 29℃ | WT | 6-12 h | 19.48 | 16.83 | 86.39 | 9.45 |
| **w1118-12-24 h-embryo-29-rep1** | 29℃ | WT | 12-24 h | 18.93 | 16.44 | 86.85 | 6.96 |
| **w1118-12-24 h-embryo-29-rep2** | 29℃ | WT | 12-24 h | 19.70 | 16.95 | 86.03 | 7.69 |
| **uKO2-0-2 h-embryo-25-rep1** | 25℃ | Mutant | 0-2 h | 16.22 | 14.94 | 92.12 | 4.27 |
| **uKO2-0-2 h-embryo-25-rep2** | 25℃ | Mutant | 0-2 h | 16.23 | 14.91 | 91.91 | 4.15 |
| **uKO2-2-6 h-embryo-25-rep1** | 25℃ | Mutant | 2-6 h | 16.80 | 14.50 | 86.31 | 7.53 |
| **uKO2-2-6 h-embryo-25-rep2** | 25℃ | Mutant | 2-6 h | 19.34 | 16.58 | 85.71 | 7.61 |
| **uKO2-6-12 h-embryo-25-rep1** | 25℃ | Mutant | 6-12 h | 16.17 | 14.20 | 87.80 | 8.49 |
| **uKO2-6-12 h-embryo-25-rep2** | 25℃ | Mutant | 6-12 h | 16.34 | 14.36 | 87.90 | 8.11 |
| **uKO2-12-24 h-embryo-25-rep1** | 25℃ | Mutant | 12-24 h | 16.85 | 14.35 | 85.20 | 11.33 |
| **uKO2-12-24 h-embryo-25-rep2** | 25℃ | Mutant | 12-24 h | 16.88 | 14.25 | 84.44 | 12.32 |
| **w1118-0-2 h-embryo-25-rep1** | 25℃ | WT | 0-2 h | 17.29 | 15.74 | 91.06 | 5.27 |
| **w1118-0-2 h-embryo-25-rep2** | 25℃ | WT | 0-2 h | 19.53 | 17.83 | 91.26 | 5.06 |
| **w1118-2-6 h-embryo-25-rep1** | 25℃ | WT | 2-6 h | 19.23 | 16.85 | 87.62 | 7.76 |
| **w1118-2-6 h-embryo-25-rep2** | 25℃ | WT | 2-6 h | 17.22 | 14.86 | 86.27 | 9.67 |
| **w1118-6-12 h-embryo-25-rep1** | 25℃ | WT | 6-12 h | 19.04 | 16.02 | 84.15 | 11.76 |
| **w1118-6-12 h-embryo-25-rep2** | 25℃ | WT | 6-12 h | 17.59 | 15.00 | 85.31 | 10.71 |
| **w1118-12-24 h-embryo-25-rep1** | 25℃ | WT | 12-24 h | 16.25 | 14.03 | 86.35 | 10.29 |
| **w1118-12-24 h-embryo-25-rep2** | 25℃ | WT | 12-24 h | 16.23 | 14.08 | 86.73 | 9.37 |
