# Supplementary material for: Upstream open reading frames buffer translational variability during Drosophila evolution and development
Source: eLife. 2025 Jun 6;14:RP104074. doi: 10.7554/eLife.104074 (PMC12143884; doi:10.7554/eLife.104074)
Supplement: Supplementary file 5. [file elife-104074-supp5.docx]

**Supplementary File 5.** **Numbers of genes showing different magnitudes of TE changes between uORFs and CDS at the interspecific level, *H. sapiens* and *M. mulatta*.**

| **Tissues** | **# of expressed uORFs** | **β_u_ ≠ 1**  **(%)** | **# of expressed CDSs** | **β_c_ ≠ 1**  **(%)** | **uORF-CDS pairs with β_u_ > 1** | | | **uORF-CDS pairs with β_u_ < 1** | | |
| --- | --- | --- | --- | --- | --- | --- | --- | --- | --- | --- |
|  |  |  |  |  | **Total** | **γ > 1** | **γ < 1** | **Total** | **γ > 1** | **γ < 1** |
| **Brain** | 7,380 | 80  (1.08) | 15,086 | 507  (3.36) | 51 | 0 | 27 | 29 | 14 | 0 |
| **Liver** | 4,429 | 28  (0.63) | 13,246 | 149  (1.24) | 10 | 0 | 4 | 18 | 10 | 0 |
| **Testis** | 7,384 | 53  (0.72) | 15,134 | 272  (1.80) | 33 | 0 | 9 | 20 | 5 | 0 |

Only uORFs and CDSs with an mRNA RPKM > 0.1 in both *H. sapiens and* *M. mulatta* were considered in each sample pair in the analysis. $\beta_{u}$ = ${TE}_{uORF, macaque}$/${TE}_{uORF, human}$ , is the fold change in TE_uORF_ in *M. mulatta* relative to *H. sapiens* for each sample. $\beta_{c}$ = ${TE}_{CDS, macaque}$/${TE}_{CDS, human}$, is the fold change in TE_CDS_ in *M. mulatta* relative to *H. sapiens* for each sample. $\gamma$ =$\beta_{c}/\beta_{u}$. For each CDS-uORF pair, $\beta_{u}$>1 and $\gamma$ < 1 or $\beta_{u}$<1 and $\gamma$ > 1 means that the magnitude of TE change is lower for a CDS than a uORF. The statistical significance of $\beta_{c}$, $\beta_{u}$, and $\gamma$ were all determined at an FDR < 0.05.
